# High-Dimensional Analysis of Immune Cell Composition Predicts Periprosthetic Joint Infections and Dissects Its Pathophysiology

**DOI:** 10.3390/biomedicines8090358

**Published:** 2020-09-17

**Authors:** Maximilian F. Korn, Richard R. Stein, Andreas Dolf, Farhad Shakeri, Andreas Buness, Cäcilia Hilgers, Werner Masson, Sascha Gravius, Hendrik Kohlhof, Christof Burger, Dieter C. Wirtz, Thomas M. Randau, Frank A. Schildberg

**Affiliations:** 1 Clinic for Orthopedics and Trauma Surgery, University Hospital Bonn, 53127 Bonn, Germany; kornmaximilian@gmx.net (M.F.K.); caecilia.hilgers@ukbonn.de (C.H.); werner.masson@ukbonn.de (W.M.); Sascha.Gravius@umm.de (S.G.); hendrik.kohlhof@ukbonn.de (H.K.); christof.burger@ukbonn.de (C.B.); dieter.wirtz@ukbonn.de (D.C.W.); thomas.randau@ukbonn.de (T.M.R.); 2 cBio Center, Department of Data Science, Dana-Farber Cancer Institute, Boston, MA 02115, USA; richard.stein@novartis.com; 3 Institute of Experimental Immunology, University Bonn, 53127 Bonn, Germany; andreas.dolf@uni-bonn.de; 4 Institute for Genomic Statistics and Bioinformatics, Medical Faculty, University of Bonn, 53127 Bonn, Germany; farhad.shakeri@uni-bonn.de (F.S.); andreas.buness@uni-bonn.de (A.B.); 5 Institute for Medical Biometry, Informatics and Epidemiology, Medical Faculty, University of Bonn, 53127 Bonn, Germany; 6 Core Unit for Bioinformatics Data Analysis, Medical Faculty, University of Bonn, 53127 Bonn, Germany; 7 Department of Orthopaedics and Trauma Surgery, University Medical Center Mannheim of University Heidelberg, 68167 Mannheim, Germany

**Keywords:** periprosthetic joint synovial fluid, periprosthetic joint infection, aseptic implant failure, immune cell infiltrate, myeloid-derived suppressor cells

## Abstract

Accurate diagnosis of periprosthetic joint infections (PJI) is one of the most widely researched areas in modern orthopedic endoprosthesis. However, our understanding of the immunological basis of this severe complication is still limited. In this study, we developed a flow cytometric approach to precisely characterize the immune cell composition in periprosthetic joints. Using high-dimensional multi-parametric data, we defined, for the first time, the local immune cell populations of artificial joints. We identified significant differences in the cellular distribution between infected and non-infected samples, and revealed that myeloid-derived suppressor cells (MDSCs) act as potential regulators of infiltrating immune cells in PJI. Further, we developed an algorithm to predict septic and aseptic samples with high sensitivity and specificity, that may serve as an indispensable addition to the current criteria of the Musculoskeletal Infection Society. This study describes a novel approach to flow cytometrically analyze the immune cell infiltrate of joint fluid that not only improves our understanding of the pathophysiology of PJI, but also enables the development of a novel screening tool to predict infection status. Our data further suggest that pharmacological targeting of MDSCs represents a novel strategy for addressing PJI.

## 1. Introduction

Periprosthetic joint infections (PJI) are among the most severe complications in modern endoprosthesis [1,2]. During revision of chronically infected endoprosthesis, the implant must first be removed, and a new joint can only be implanted after 2–6 weeks of treatment with antibiotics if signs of infection have subsided [3,4]. Stringent restrictions for the patient undergoing revision of the contaminated implant are accompanied by enormous expense, with the costs of treatment for PJI clearly exceeding that of primary implantation without complications by three to four times [5].

The accurate diagnosis of PJI remains a significant challenge, mainly because of the lack of a “gold-standard” for the detection of the potential presence of bacteria in the joint [6]. The absence of an optimal screening parameter is clearly reflected by the variety of multifactorial criteria applied in the past couple of years: From the criteria of the Infectious Diseases Society of America (IDSA) to those of the Musculoskeletal Infection Society (MSIS) in 2018, the goal has been to capture the complexity of PJI as sensitively and specifically as possible by using a combination of different factors to diagnose the disease accurately [7].

Although the diagnostic pattern provided by these definitions would be suitable for use in daily clinical practice, using these exact cut-off values as rigid thresholds can potentially cause problems; Christensen et al. report that the MSIS parameter of the white blood cell count (WBC) and the percentage of granulocytes (PMN%) are only applicable to implants inserted at least three to six months previously. For implants with a shorter duration between implantation and measurement of WBC and PMN%, these authors report a false positive rate of up to 25%, and recommend significantly higher cut-off values [8].

Additional parameters, such as C-reactive protein (CRP) levels, erythrocyte sedimentation rate (ESR), and leucocyte count in the blood, are sensitive but less specific markers that are elevated in several diseases, and strongly affected by age, gender, and comorbidities [9]. The microbiological analysis, often referred as the “gold-standard” in the PJI diagnostic, often yields false negative results, from 5% up to 34% of the samples [10]. The reason for this high percentage is often biofilm association or low virulence of the respective bacteria, which makes them exceedingly difficult to maintain in culture and detect [11]. Furthermore, the “time factor” should be considered; certainly, it is inadvisable to wait several days for microbiology or pathology results in cases of acute joint infections. For an optimal outcome, both diagnosis and treatment must be performed as soon as possible.

In terms of additional parameters that may be applied, the use of serous and synovial biomarkers is currently paramount to reinforce the already used diagnostic criteria. Various groups, such as Deirmengian et al. [12], are currently testing different biomarkers for their sensitivity and specificity when used along with different cut-off values. Although certain markers, such as leucocyte esterase (LE) and α-defensin, are already established as part of international diagnostic criteria [13], others are currently being discussed and need further investigation [12]. This lack of studies is not restricted to soluble factors; the immune cell composition has also only been superficially investigated. To date, there is no existing cellular classification that describes the immune cell composition of synovial fluid in the context of PJI. Although this may have been attributable to technical limitations in the past, recent developments in the field of flow cytometry and its application in the clinical setting have paved the way for novel multiparametric analysis of different sample types.

Therefore, the goal of this study was to use high-dimensional flow cytometric data to analyze the immune infiltrate of joint fluid in order to improve our understanding of the pathophysiology of PJI, and to potentially develop a prototype of a novel screening tool to potentially predict infection status. For this purpose, knee and hip aspirates from patients with either septic or aseptic prosthetic loosening were collected and flow cytometrically analyzed, and it was hypothesized that there should be a significant difference in the immune cell composition of infected and non-infected joint aspirates. These differences have the potential to fundamentally advance our understanding of the immune dynamics during joint infections.

## 2. Experimental Section

### 2.1. Patients, Sample Collection, and Classification

Synovial fluid samples were acquired by the same clinical practitioner via strict aseptic aspiration from the affected joint. Joint aspirates were used to determine infection status and thereby to differentiate aseptic loosening from infection. To avoid differences generated by different aspiration settings, only intraoperative samples during revision surgery were included. This resulted in an inclusion of 50 out of 77 recruited patients. Only patients with current knee or hip arthroplasties were considered. Native joints and sine-situations without prosthesis, such as girdlestone hips or inlaying spacers, were excluded. This study was approved by the local institutional review board (University of Bonn ethics committee) and was performed in accordance with the declaration of Helsinki.

For sample classification, a slightly modified version of the already established MSIS criteria was used, following a defined set of major and minor criteria: The major criteria were as follows: (1) A sinus tract with access to the joint and (2) two positive microbiological cultures with the same pathogen. The minor criteria were as follows: (1) Elevated WBC count in joint aspirate (>3000/μL), (2) elevated PMN% in joint aspirate (>80%), (3) elevated serum CRP (>10 mg/L), (4) single positive microbiological culture (including sonicated parts of the implant, tissue samples, and joint aspirate), and (5) positive pathology (classification by Morawietz et al. [14]; grade ll or lll). Patients with either one positive major criterion or three positive minor criteria were considered as septic loosening (infected); all other patients were defined as aseptic loosening (non-infected). Based on this scheme and depending on the individual combination of minor and major criteria, not all collected classification parameters were necessary for all patients. In borderline cases, also the clinical intraoperative findings of the surgeon were used as a classifier.

### 2.2. Sample Preparation

The collected joint aspirate was divided into supernatant and cells by centrifugation for 10 min at 1200 rpm. The resulting pellet was resuspended in MACS buffer (99 mL PBS (Life Technologies, Darmstadt, Germany), 1 mL FBS, and 0.06 g EDTA (Carl Roth GmbH, Karlsruhe, Germany) and filtered through a cell strainer (FALCON cell strainer 70 μm) to eliminate any remaining joint debris. Erythrocytes were lysed using 5 mL lysis buffer (16.58 g ammoniochloride (Carl Roth GmbH), 2 g potassium hydrogencarbonate (Carl Roth GmbH), 74.4 mg EDTA (Carl Roth GmbH), and 2000 mL distilled water). Isolated cells were counted and subsequently transferred into freezing medium (50% DMEM low glucose, pyruvate (Life Technologies), 40% FBS Superior (Gibco), 10% dimethylsulfoxide (Carl Roth GmbH), and stored at −150 °C. Staining of surface molecules and flow cytometric analysis was performed with thawed cells out of cryopreservation to minimize incubation time and cellular stress.

In addition to the processing in the laboratory, all samples underwent microbiological, pathological, and clinical biochemical analysis. Microbiologic analysis included cultures and PCR from intraoperatively acquired membrane tissue, synovial fluid, and sonicated prosthesis components, if applicable, to identify the respective pathogen. Classification of membrane samples according to Morawietz et al. [14] was performed using histopathological examination. Clinical biochemical analysis included the quantification of synovial WBCs and polymorphonucleocyte (PMN) count, in addition to the analysis of serum CRP levels.

### 2.3. Flow Cytometric Analysis

For flow cytometric analysis, frozen samples were thawed; then, cells were resuspended in PBS with 1% FBS/2 mM EDTA and stained with fluorescently labelled antibodies for 20 min. Unstained and FMO (fluorescence minus one) samples were used as controls. Markers and clones were selected based on published data or based on previous experiments at our laboratory [15,16,17,18]. Titration experiments were performed to define the optimal antibody concentrations resulting in distinct staining with minimal spillover. Antibodies were purchased from BioLegend (San Diego, CA, USA), Thermo Fisher Scientific (Karlsruhe, Germany), and BD Bioscience (Heidelberg, Germany), and fluorochromes were selected using the Multicolor Panel Selector from BioLegend (Table S1). Flow cytometry data were acquired on a LSRFortessa analyzer 10 flow cytometer (BD Biosciences). Laser calibration was performed using CS&T beads (BD Biosciences) in all experiments, and FCS files were analyzed using FlowJo software (BD Biosciences). Gating strategies were adapted from previously published studies [15,16,19] using unstained and FMO samples. In some cases, because of the nature of the heterogenic patient material, batched gates required manual correction to compensate for slight differences between samples. Only samples with at least 400 single and living cells were included in the analysis.

### 2.4. Statistics, Logistic Regression Analysis, and Classification Algorithms

Data were collected in Microsoft Excel (Microsoft Corporation, Richmond, VA, USA), and statistical analysis was carried out using IBM SPSS Statistics 25 (IBM Corporation, Armonk, NY, USA) and GraphPad Prism 7 (GraphPad Software, La Jolla, CA, USA). Between-group analysis was performed using the Mann–Whitney U test. Cut-off ratios were calculated and chosen by ROC analysis and Youden Index. Significance levels are marked as * *p* < 0.05, ** *p* < 0.01, *** *p* < 0.001, and **** *p* < 0.0001.

Based on the immune cell proportions and with the help of R, a logistic regression model, logit *p* = β_0_ + β_1_x_1_ + ⋯ + β_k_x_k_ [20], was used to generate a reduced predictive infection classification model. In this model β_i_ were the parameters of the model and x_i_ were immune cell concentrations of the patients. First, we put all variables into the model, followed by a rough selection based on the *p*-value so that the logistic regression model converges. Then, we identified the most relevant variables on this preselected subset.

In addition to the logistic regression analysis, we used other standard classification algorithms, implemented in the mlr software package [21], to estimate the misclassification error. The following 5 different classifiers were applied to the data set: Support Vector Machines (ksvm) [22], Decision Tree (rpart) [23], Linear Discriminant Analysis (lda) [24], Random Forest (randomForest) [25], and Conditional Inference Trees (ctree) [26]. To evaluate the performance of the classification methods, we chose the mean misclassification error (mmce). To obtain an unbiased mmce, a nested resampling method with 10-fold cross-validation in both inner and outer loops was applied. The inner loop was used to reduce the overfitting of hyperparameter tuning. For hyperparameter tuning, we used the default parameter space configurations as implemented in the corresponding functions in the mlr package. However, for the ksvm and rpart, we moderately extended the parameter space.

## 3. Results

To identify the immune cells that occur in infected periprosthetic joints, we divided 50 of our previously collected samples into a non-infected group, representing patients with aseptic loosening, and an infected group, representing patients with septic loosening. Patients with either a sinus tract with access to the joint or a double-positive microbiological result were classified as infected. Additionally, patients were considered as infected if three out of five minor criteria were met: Elevated CRP level (>10 mg/L), elevated synovial leucocytes (>3000/μL), elevated percentage of synovial granulocytes (>80%), a single positive microbiological culture or positive PCR result for explanted parts or synovial fluid, and a pathological report of a type II or III Morawietz membrane [14]. Patients who did not meet these criteria were considered non-infected.

**Figure 1:**
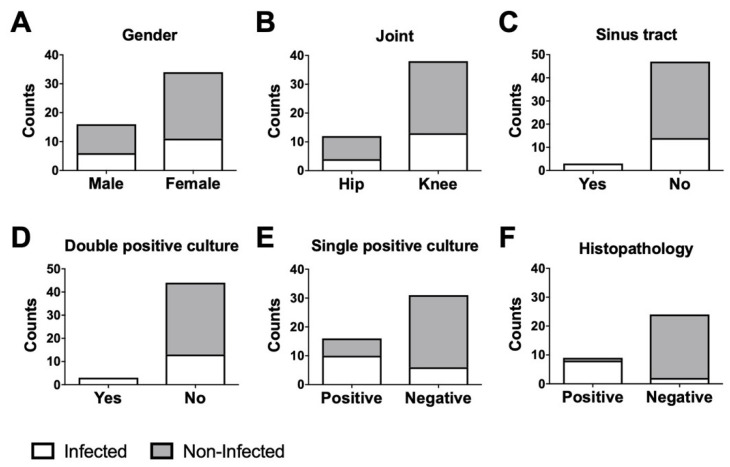
Demographic features of the non-infected and infected groups. Distribution of (**A**) gender, (**B**) joint type (hip or knee), (**C**) patients with or without joint-communicating sinus tract, (**D**) double-positive microbiological result, (**E**) single-positive microbiological result, and (**F**) positive histopathology report in the non-infected and infected groups.

According to our modified MSIS criteria, 17 patients were considered infected and 33 non-infected. The infected group consisted of six men and 11 women with a mean age of 72.2 years; the non-infected group consisted of 10 men and 24 women with a mean age of 65.6 years (Figure 1A).

The ratio of knees and hips in each group were identical: There were four hips and 13 knees in the infected group, and eight hips and 26 knees in the non-infected group (Figure 1B). Three infected patients showed a sinus tract with access to the joint (Figure 1C), and three infected patients showed a double positive microbiological report (Figure 1D). Further, 10 infected and six non-infected patients presented with a single positive microbiological culture (Figure 1E). In two infected and five non-infected cases, a microbiological report was not available. Eight infected and one non-infected patient showed positive pathological results (Figure 1F). In seven infected and 10 non-infected cases, a pathology report was not available.

**Figure 2:**
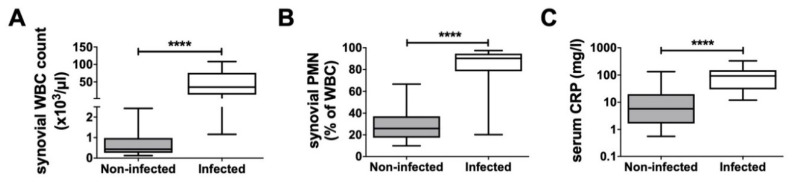
Clinical data for the patient cohort. (**A**) Distribution of synovial leucocytes (non-inf. *n* = 20, inf. *n* = 13), (**B**) proportion of neutrophil granulocytes among synovial leucocytes (non-inf. *n* = 20, inf. *n* = 13) and (**C**) serum C-reactive protein (CRP) levels (non-inf. *n* = 30, inf. *n* = 15) in the non-infected and infected groups. **** *p* < 0.0001.

The infected group had a mean synovial WBC count of 89,322.8 cells/μL, a synovial PMN% of 81.0%, and a serum CRP level of 111.4 mg/L. In comparison, the non-infected group had a WBC count of 669.1 cells/μL, an PMN% of 28.5%, and a serum CRP level of 17.2 mg/L (Figure 2). In four infected and 13 non-infected cases, the synovial WBC count and PMN% were not analyzed. For two infected and three non-infected patients, reports of their serum CRP level were not available.

**Figure 3:**
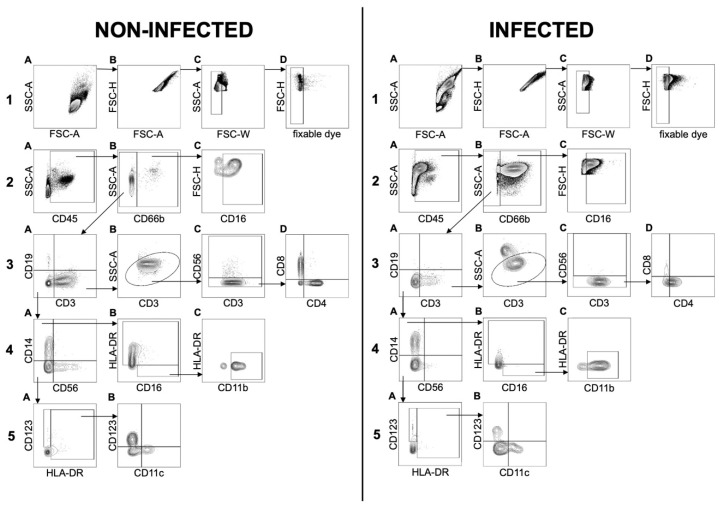
Flow cytometry analysis of leucocyte populations present in the synovial fluid of non-infected (**left**) and infected (**right**) patients. The gating strategy for all samples was performed as following: General cell distribution of the whole sample is shown in (**1A**). First, exclusion of doublets (**1B**,**C**) using FSC-A/FSC-H and FSC-W/SSC-A and dead cells (**1D**) using fixable dye. Further gating for CD45 enabled selection of only leucocytes for further investigation (**2A**). The leucocytic population was divided into CD66b^+^ neutrophil and eosinophil granulocytes and CD66b^−^ cells (**2B**). Neutrophil and eosinophil granulocytes were identified based on the expression of CD16 (**2C**). All CD66b^−^ cells were gated for the expression of CD3 and CD19 to distinguish potential T cells from B cells (**3A**). SSC in combination with CD3 expression was used to minimize contamination of the previously gated CD3^+^ population (**3B**). Expression of CD56 was used to detect NKT cells (**3C**). All other CD3^+^ cells were gated for CD4 and CD8 to isolate T_h_ and T_k_ cells (**3D**). Boolean gating of non-gated cells in 3B and all CD3^−^ and CD19^−^ cells was performed. The resulting population was analyzed for CD56 and CD14 expression to identify NK cells and monocytic cells, respectively (**4A**). The monocytic population was divided into HLA-DR^+^ monocytes and HLA-DR^−^ and CD16^+^ potential mMDSCs (monocytic myeloid-derived suppressor cells) (**4B**). Potential mMDSCs were identified using CD11b (**4C**). The remaining cells were gated for HLA-DR and CD123 expression to distinguish basophile granulocytes from potential dendritic cells (**5A**). Myeloid (mDCs) and plasmacytoid dendritic cells (pDCs) were identified by expression of CD11c (**5B**).

Our newly established flow cytometry panel allowed the analysis of the general leucocyte cell count as well as 14 different markers to distinguish between 11 cell types in total: Neutrophils, eosinophils, basophils, B cells, T_k_ cells, T_h_ cells, NKT cells, monocytes, NK cells, dendritic cells (DCs) and monocytic myeloid-derived suppressor cells (mMDSCs) (Figure 3). After exclusion of debris, doublets, and dead cells using FSC-A/FSC-H, FSC-W/SSC-A, and fixable dye, respectively, the leucocyte population was divided into two major subsets: CD66b^+^ granulocytes and CD66b^−^ cells. Because the total number of cells in each sample was different, relative cell numbers were used to compare the composition of the cell populations of infected and non-infected samples. Both groups were statistically analyzed to determine potential differences in their general cell composition.

**Figure 4:**
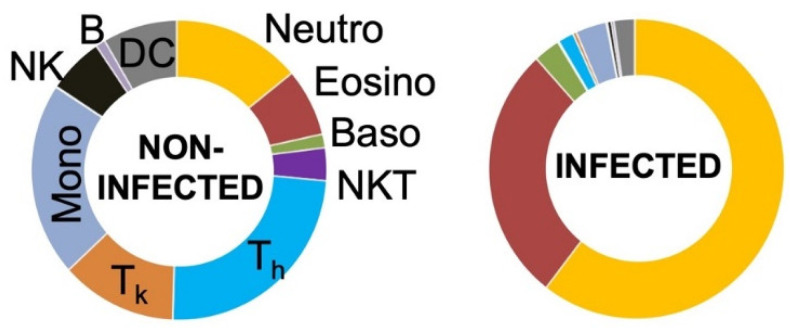
Distribution of identified subpopulations in relation to the overall CD45^+^ leucocyte count. The massive infiltration of neutrophil granulocytes in infected joints resulted in shifted leucocytic subtype proportions and significance for granulocytes, T cells, monocytes, natural killer (NK) cells, B cells, and dendritic cells (DCs).

Because CD45 was used as the general marker for leucocytes, our first approach was to analyze all populations in relation to the overall CD45^+^ count (Figure 4). A generally higher CD45 cell count was detected in infected samples, indicating the recruitment of leucocytes to the site of inflammation. Joint injuries, rheumatoid arthritis, and other pathological conditions have been reported to be associated with increased cytokine levels and cellular synovial components [27]. Our study showed that granulocytes are the major cell population infiltrating the joint (Figure 4).

Although significant differences between non-infected and infected joint fluids were noticed, the massive increase in CD45^+^ cells in infected cases led to a shift in the proportions of all other subpopulations, masking potential differences in specific cell types. Therefore, leucocytes were divided into granulocytic and non-granulocytic cells for more detailed analyses.

**Figure 5:**
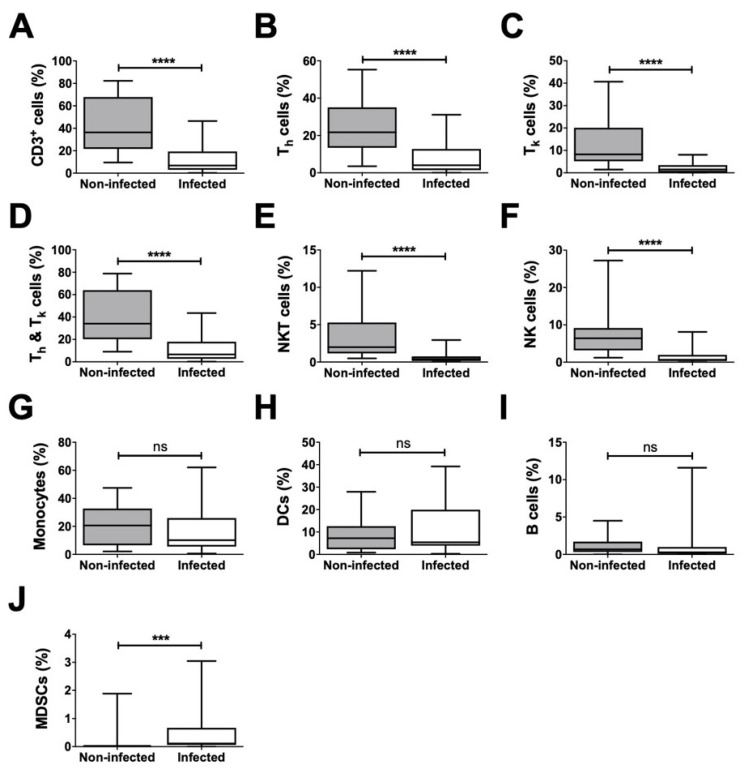
Detailed distribution of identified subpopulations in relation to all non-granulocytic cells. Statistical differences in ratios of the non-granulocyte cell population (**A**–**J**) between non-infected and infected samples; after the exclusion of granulocytes, the infected samples were found to have a significantly lower average of cell count ratios for CD3^+^, T_h_ cells, T_k_ cells, NKT cells, and NK cells. Only the proportion of MDSCs was increased in infected synovial fluid relative to that in non-infected samples. *** *p* < 0.001, **** *p* < 0.0001, ns: non-significant.

Analysis of all non-granulocytes revealed a significantly lower ratio for CD3^+^ cells, including their T helper (T_h_) and T killer (T_k_) cell subsets, as well as for NKT and NK cells in infected samples in comparison to that in non-infected samples (Figure 5). Further, the proportion of the combined population of T_h_ and T_k_ cells among the non-granulocytes was significantly decreased in infected joint aspirates (Figure 5). However, the proportions of monocytes, DCs, and B cells were not significantly different. Interestingly, mMDSCs were the only non-granulocytic cells that were significantly increased in infected samples.

**Figure 6:**
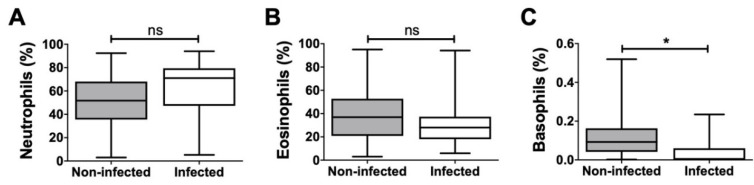
Proportion of granulocyte subpopulations in relation to total granulocytes. Percentage of (**A**) neutrophils, (**B**) eosinophils and (**C**) basophils for both non-infected and infected samples are shown. Only basophils showed a significant difference between non-infected and infected samples. * *p* < 0.05, ns: non-significant.

**Figure 7:**
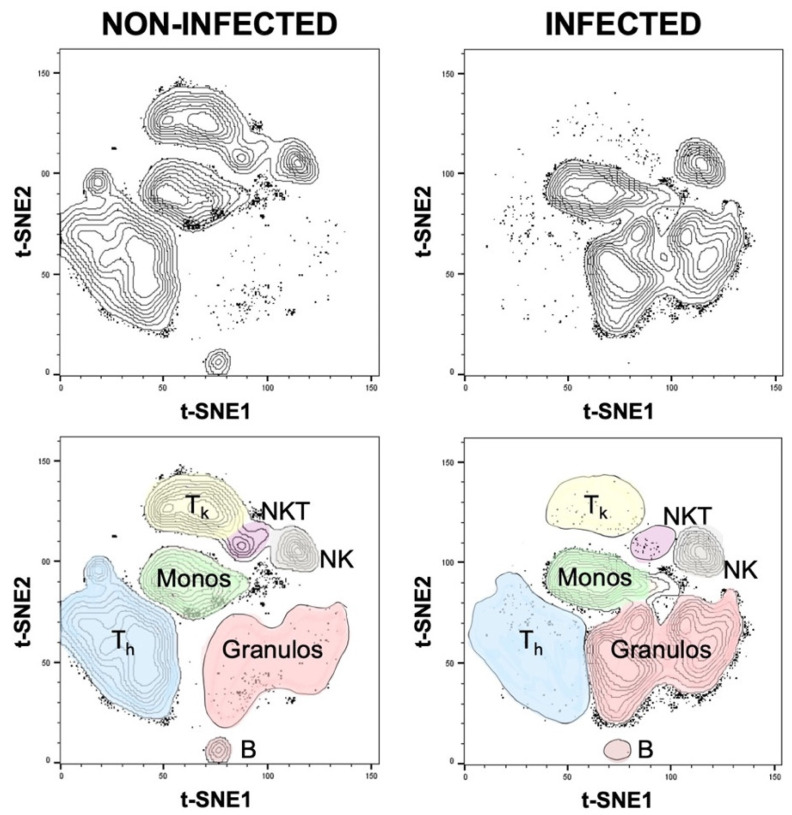
t-SNE plots for the non-infected and infected synovial cell populations.

Additionally, the proportion of neutrophilic, eosinophilic, and basophilic granulocytes in relation to the overall granulocyte count was analyzed, and significant differences were detected only in the case of basophilic granulocytes (Figure 6). These differences were also confirmed using t-SNE plots, a popular tool for visualizing high-parameter single-cell data in biaxial plots. The generation of t-SNE plots nicely recapitulated our findings (Figure 7). They not only confirmed how different the cellular composition of non-infected and infected aspirate samples is, but they also intuitively illustrate the decreased proportion of T_h_, T_k_, and NKT cells as well as an increased proportion of granulocytes in the infected group. The depicted t-SNE plots basically represent an “immunological fingerprint” of the cellular composition of the whole joint aspirate (Figure 7) and give a great overview of cellular changes between non-infected and infected join aspirates.

In summary, using this flow cytometric approach, we defined, for the first time, the local immune cell populations of artificial joints. We identified significant differences in the cellular distribution between infected and non-infected samples, and revealed hints that myeloid-derived suppressor cells (MDSC) could act as potential regulators of the immune cell infiltrate in PJI. The identified cellular changes from our pilot data not only improve our understanding of the pathophysiology of PJI but are also useful for the development of a potential novel screening tool to predict infection status.

**Figure 8:**
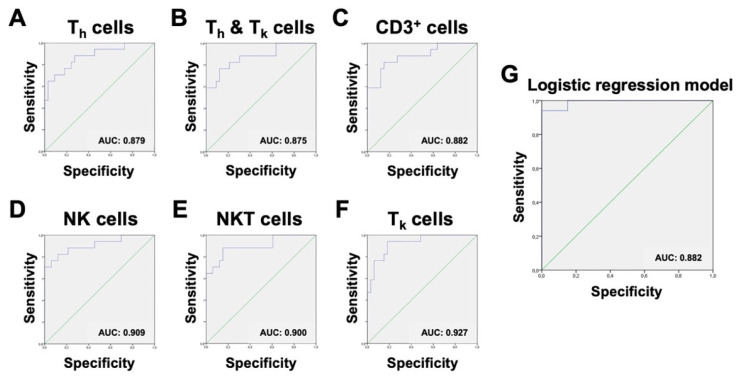
Results of ROC analysis of the most significant non-granulocytic ratios. Ranking from (**A**–**F**) shows the graphs in ascending order of their respective Youden-index. Areas under the curve (AUCs) are displayed at the bottom right of the diagram. Graph (**G**) shows the ROC curve of the logistic regression model, representing an algorithm combining the abundance of NK cells, CD3^+^ cells, monocytes, and neutrophil granulocytes.

Therefore, our next goal was to establish cut-off ratios to make these potential new test parameters practicable and comparable. ROC analysis was performed on every significant cell ratio within the non-granulocytes. ROC curves were ranked based on their area under the curve (AUC). A higher AUC was considered to offer a greater potential for discrimination between the infected and non-infected group. ROC curves with an AUC of less than 0.8 were excluded. To objectify and rank the cut-off ratios, the Youden-Index was used. All relevant ROC graphs are shown in Figure 8. In short, three cell ratios were identified to have adequate sensitivity and specificity for consideration as additional parameters for the already established MSIS criteria. Because a combination of these parameters is only sufficient if the parameters are not dependent on each other, it is important to take into account that the chosen populations are not subtypes of each another. In this study, the ratios of NK cells, NKT cells, and T_k_ cells within the non-granulocyte fraction were found to be most suitable as a useful addition to the MSIS criteria, because they represent a combination of sensitive and specific parameters and are gated independently.

In addition to the previously described ROC analysis, logistic regression was performed to obtain a predictive algorithm. The aim was to model the MSIS criterion-based infection classification using data from immunologic patient profiles, without inclusion of data already used for the definition of the MSIS classifier. The normalized FACS-derived cell numbers of subtypes of non-granulocytic cells (NK cells, CD3^+^ T cells, B cells, DCs, monocytes, MDSCs), and subtypes of granulocytic cells (basophiles, eosinophils, and neutrophils) served as predictor variables. The response variable was given by the MSIS-derived infection classification, which was denoted by “1” if the sample was classified as infected, and “0” otherwise. In order to exclude information regarding the total number of cells per sample, which is used in the definition of the MSIS infection classifier, we normalized the subtype counts by the number of granulocytic or non-granulocytic cells.

Based on the above analyses, we constructed logistic regression models from relevant variable subsets and proposed a reduced predictive infection classification model. To predict the binary outcomes of infected vs. non-infected samples from flow cytometric data, we applied a logistic regression model, logit *p* = β_0_ + β_1_x_1_ + ⋯ + β_k_x_k_ [20], where β_i_ are the parameters of the model and x_i_ are relevant immune cell concentrations of the patients. For model training, we included all 50 patient samples: 17 infected and 33 non-infected, respectively. In order to ensure convergence, logistic regression models were fitted with R function *glm* to one covariate at a time, and variables with *p*-values of less than 0.25 were selected. This excluded the variables of B cells and eosinophils from the set of predictor variables, and the resulting full model included seven predictor variables. In order to select the most relevant subset of observables, we applied forward variable selection to the preselected seven variables based on the Bayesian Information Criterion with the constant null model, using the R function *step*. The resulting best model consisted of a subset of four predictor variables containing the relative abundances of NK cells, CD3^+^ T cells, monocytes, and neutrophils. The model coefficients β_i_ were 1.377 for the intercept, −92.711 for NK cells (*p*-value: 0.0460), −17.524 for CD3^+^ T cells (*p*-value: 0.0619), −12.365 for monocytes (*p*-value: 0.0606), and 15.043 for neutrophils (*p*-value: 0.0361). The significance of the full model compared to a constant null model was 2.1 × 10^−10^.

**Figure 9:**
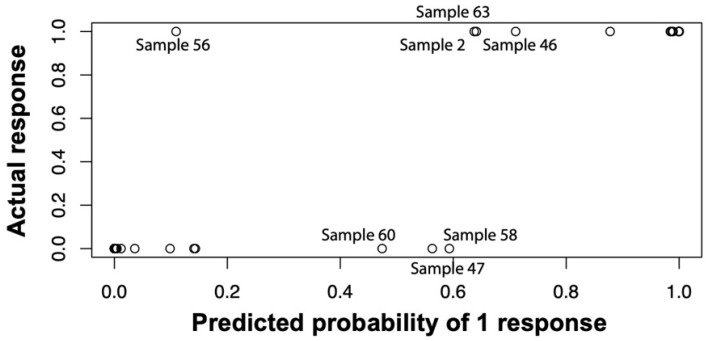
Comparison of predicted probability of infection by our logistic regression algorithm versus the actual response as defined by our modified Musculoskeletal Infection Society (MSIS) criteria. “0” indicates non-infected samples and “1” indicates infected samples. The “actual response” was based on our modified MSIS classification. Highlighted samples 2, 46, 47, 56, 58, and 60 were borderline cases whose clinical presentation was analyzed for the purpose of assignment to either the infected or non-infected group. Based on this clinical appearance, a cut-off of 0.5185 was chosen, which identified samples 47, 56, and 58 as ill-classified.

To assess the general ability of our logistic regression model to discriminate infected from non-infected samples, we applied the above-described model to the full training dataset and saw sufficient separation of infected vs. non-infected patients with ill-classified samples 47, 56, and 58 for 0.5185 as the cut-off probability of infected and non-infected (Figure 9). The expected misclassification error of the logistic regression approach was estimated by a leave-one-sample-out cross validation. In detail, we performed the above-described forward variable selection on every subset of 49 of the 50 patient samples and predicted whether the hold-out patients were infected or not based on the single-patient immune profile. The resulting misclassification error was found to be 18.2% when 0.5 was used as cut-off probability, and 16.0% when 0.8 was used. At a cut-off of 0.5185, ROC analysis of the resulting probabilities for our algorithm showed a sensitivity of 94.1% and a specificity of 93.9% (Figure 8).

**Figure 10:**
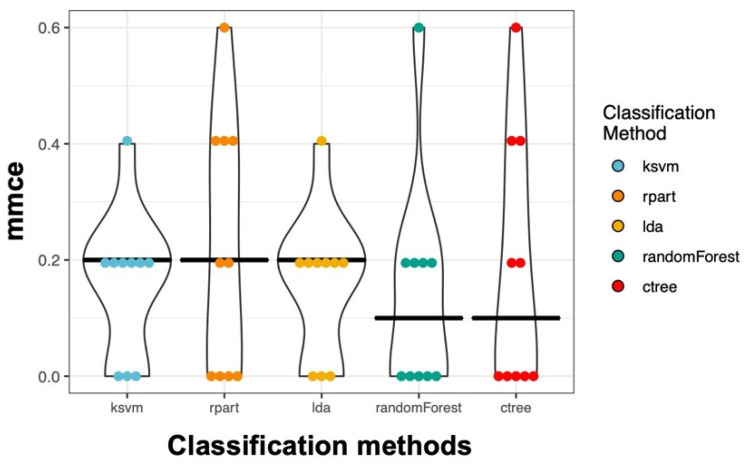
Mean misclassification errors for the five classification methods. The mean misclassification errors (mmce) for the five classification methods and 23 predictor variables (kvsm: Support Vector Machines, rpart: Decision Tree, lda: Linear Discriminant Analysis, randomForest: Random Forest, ctree: Conditional Inference Trees) are depicted as violin plots for all resampling iterations. Resampling strategy: 10-fold cross-validation. Each point represents an iteration.

In addition to the logistic regression analysis, we used methods that can also deal with a very large number of variables. Therefore, we selected all useful parameters (Table S2) from our flow cytometry data and performed an unbiased analysis. In detail, we applied 5 different classification methods. Using a 10-fold nested cross-validation, we obtained on average a 17.2% mean misclassification error (mmce) across all classifiers and all iterations. The Random Forest performed best with an overall mmce of 14.0% (Figure 10).

Overall, these results are in agreement with those obtained by the logistic regression approach. In summary, 23 predictor variables were used to predict the infection status, and all of these predictor variables were derived from flow cytometry data only. To further evaluate the performance of the classification methods in a scenario more similar to real clinical application, we added one more predictor variable: The percentage of non-granulocytes. When using these 24 predictor variables, which were all based on flow cytometry data, the overall mmce estimate dropped to 7.2% (Figure S1), which was kind of expected as non-granulocytes are part of the MSIS definition of the joint infection status. This combination of variables might be an alternative way to predict joint infection in the clinical setup.

## 4. Discussion

PJI is one of the most severe complications in endoprosthetic joint replacement. The advantages of the current system used to distinguish between aseptic and septic loosening lie in its high variability and multiparametric approach; however, these strengths also give rise to a key challenge: Although application of the so-called MSIS criteria requires the testing of as many parameters as possible, in clinical outpatient settings, specific tests are often not performed to save time. The incubation periods of less virulent pathogens, such as *Propionibacterium acnes*, may be up to 14 days long [28]. The same challenges arise in relation to the pathological report, which may also take a few days for sufficient results to be obtained in clinical practice. Understandably, patients are difficult to classify: The unavailability of some necessary results may limit the utility of standard operating procedures.

To enhance the diagnostic accuracy for PJI, it is necessary to use new technologies to overcome current limitations of culture, molecular, protein-based, and imaging techniques [29]. In our study, a flow cytometric approach was applied to achieve high-dimensional multiparametric resolution of immune cell populations, and to potentially establish a pipeline to generate fast and reliable results. In cases where some MSIS parameters may not be analyzed because of time issues, a rapid and short test may help to fill the diagnostic gap.

Van Landuyt et al. reported that flow cytometry is an attractive application for synovial tissue analysis, pointing out the robust nature of the data and the ability to simultaneously analyze multiple cell populations [30]. Previous studies have mainly focused on lymphocytes [31], monocytes [32], or mesenchymal progenitor cells [33] in cases of rheumatoid arthritis. Therefore, this study is the first of its kind to establish a multicolor flow cytometry panel for detailed analysis of cell populations in synovial fluid, focusing on PJI. The underlying panel allowed the analysis of eleven different cell subtypes: Neutrophils, eosinophils, basophils, B cells, NKT cells, T_h_ cells, T_k_ cells, NK cells, monocytes, monocytic myeloid-derived suppressor cells (mMDSCs), and dendritic cells (DCs). This flow cytometric approach revealed a significantly lower proportion of NKT, T_h_, T_k_, and NK cells in infected samples; mMDSCs were the only non-granulocytic cells that were significantly increased.

MDSCs are known for their immune-suppressive function: Augmented expression of arginase 1 and the related depletion of arginine results in reduced T cell function [34] and cytokine production [35,36]. Therefore, it is possible that the identified differences in immune cell proportions are due to an increased amount of MDSCs in PJI cases. We therefore hypothesized that the significant presence of MDSCs results in suppression of other immune cell subsets, such as T cells, NKT cells, and NK cells, thereby affecting their proliferation and activation characteristics. Heim et al. showed that MDSCs were the dominant cell population in tissues of PJI cases, and that their predominance resulted in reduced T cell activation. Furthermore, these authors reported significantly elevated levels of IL-10, IL-6, and CXCL1 in PJI synovial tissue due to compensatory higher cytokine production to overcome T cell suppression [37]. These findings are congruent with those of Randau et al. who identified that serum and synovial IL-6 represents a promising biomarker for PJI [9].

Focusing on MDSC subtypes, Heim et al. did not report a difference between infected and non-infected joints in terms of CD33^+^ CD14^+^ HLA-DR^−^ CD66b^−^ mMDSCs. Although our study also did not show a significant difference in the mMDSC to CD45^+^ ratio, we observed an increased mMDSC/non-granulocyte ratio. This clearly points to the relevance of MDSCs in PJI, which has been overlooked in studies to date. Interestingly, Heim et al. reported different amounts of CD33^+^ CD14^low/−^ CD66b^+^ gMDSCs in infected vs. non-infected joints [37]. Although we did not incorporate analysis of CD33 in our study, our findings still show a significant increase in CD66b^+^ cells, which include neutrophils, eosinophils, and potential gMDSCs. Although studies on MDSCs and their cellular characteristics are in their infancy, both studies generally indicate that MDSCs may play a role in PJI and hypothesize that the immunosuppressive effects of these cells may drive PJI pathophysiology. The overall picture suggests that the identified differences in immune cell proportions in this study could potentially be due to increased immune suppression in PJI as a result of the presence of larger numbers of MDSCs at the site of inflammation. It can further be hypothesized that permanent immune cell suppression by MDSCs in infected joints could lead to chronic infection by preventing effector functions, culminating in a paralyzed local immune microenvironment. Taking this assumption one step further, MDSC-targeting drugs could potentially prevent immunosuppression in the joint, and therefore represent a new tool in preventing and treating PJI. Nevertheless, with our current knowledge, this assumption is still a speculative hypothesis and requires verification. Final validation will also rely on samples from normal synovial fluid as immune cell proportions might already be changed in non-infected joints in comparison to naive joints. Further, analyzing the suppressive capacity of MDSCs in vitro, investigating MDSC biology in vivo as well as testing immune regulatory cytokines in joint aspirates will be necessary to further substantiate our hypothesis. With our current data set and discussion, we basically want to encourage further interdisciplinary research to unscramble this highly interesting avenue.

Using our data for logistic regression analysis, the abundance of NK cells, CD3^+^ cells, monocytes, and neutrophil granulocytes showed in combination a sensitivity of 94.1% and specificity of 93.9%, with only three falsely predicted samples. The reasons for false prediction could be an aseptic chronic inflammation in the joint under antibiotic treatment or multiple previous chirurgical treatment attempts, both resulting in atypical cell invasion into the joint. For our samples, the algorithm seemed to be a suitable tool for distinguishing infected from non-infected patients, showing high accuracy with low misclassification error.

To our knowledge, the present study is the first to describe the immune cell distribution in periprosthetic joint infections and aseptic loosening, and the findings may be applied to elucidate the pathophysiology of PJI as well as to define new diagnostic approaches. The most appealing advantage of our new parameters is the duration in which a result could be obtained: When transferred into a clinical setting, the time frame between joint aspiration and final result could reasonably be about 2–3 h. In particular, in cases in which sufficient time for microbiological or pathological analyses is not available, our newly defined parameters could serve as a useful addition to the already used criteria. Importantly, it is possible to perform flow cytometric analysis in parallel to a variety of other tests using only a single joint aspiration, such as testing LE, α-defensin, synovial leucocyte counts, and percentage of PMNs, which makes flow cytometry a highly valuable addition to the existing and currently discussed MSIS criteria. Given access to flow cytometers, which are present in most clinics, the overall costs per assay are quite low and mostly consist of cell culture reagents and antibodies, which makes this assay not only feasible for large clinics with associated research departments. However, a certain amount of standardization and optimization for the daily business is required.

A potential challenge in daily clinical settings, however, is the standardization of infected and non-infected samples. Even in our standardized setting, large differences in the quality and composition of the samples were observed. Because this study made use of samples that were aspirated during ongoing surgery, it cannot be fully excluded that synovial samples could be contaminated with patient blood during this process. An ideal source of samples would be aspirate from the joint before the first incision; alternatively, sample collection should be performed in an outpatient setting to prevent contamination with blood. We also would recommend discarding the first aspirated milliliter, on the basis that the needle would have had contact with skin and blood during the procedure. Further, the time between aspiration and processing of the sample should be kept as short as possible to prevent potential distortion. In addition, when isolating the corresponding immune cells from the aspirates, samples should not be centrifuged for longer than necessary as the expression of certain markers is sensitive to centrifugation time and speed; for example, CD20 and CD19 levels are reported to decrease at a certain rotation speed [38]. Purification should be performed without enzymatic treatment, because such treatment is reported to decrease the expression of markers such as CD14 on monocytes and tissue-resident macrophages [39]. Furthermore, for an optimal setup in clinical practice, a cocktail of all necessary antibodies should be provided for immediate staining after cell isolation from the synovial fluid; after staining, cells should be analyzed as soon as possible.

## 5. Conclusions

It remains challenging to distinguish PJI from aseptic loosening in joint reconstruction. Given its complexity, a deeper understanding of the individual inflammatory mechanisms in the joint is highly necessary to identify new markers for correct PJI classification. In this pilot study, we showed for the first time that immune cells other than granulocytes, which represent a known and established cellular biomarker, also play an important role in PJI. Although this pilot data set needs to be verified in a larger cohort, we went ahead and used our data for logistic regression analysis. In detail, we showed that the proportion of NK cells, CD3^+^ cells, monocytes, and neutrophil granulocytes offer a basis for the detection of PJI with exceptional sensitivity and specificity, making our combination of cellular markers a promising addition to the parameters currently used for PJI diagnosis. Additionally, the current data suggest an inhibitory interaction between leucocytes and MDSCs, resulting in different cell distributions in PJI relative to those in aseptic complications. We speculate that immune suppression by MDSCs could lead to chronic joint infections and implant failure. Taking this hypothesis a step further, we propose that pharmacological targeting of MDSCs represents a novel approach for preventing and treating PJI. In summary, flow cytometric analysis of synovial fluid appears to be a promising tool for a multifactorial diagnosis system, with the advantages of accessibility, rapid results, high accuracy, and possible outpatient application before revision surgery.

## Supplementary Materials

The following are available online at https://www.mdpi.com/2227-9059/8/9/358/s1.
