# Supplementary material for: High-Dimensional Analysis of Immune Cell Composition Predicts Periprosthetic Joint Infections and Dissects Its Pathophysiology
Source: Biomedicines. 2020 Sep 17;8(9):358. doi: 10.3390/biomedicines8090358 (PMC7554968; doi:10.3390/biomedicines8090358)
Supplement: Supplementary file 1 [file biomedicines-08-00358-s001.pdf]

Supplementary data

Marker	Color	Clone	Company	Isotype	Order number	Recommended concentration
CD3	BV510	SK7	Biolegend	Mouse IgG1,κ	344827	5 μl/10^6 cells
CD14	BV605	M5E2	Biolegend	Mouse IgG2a,κ	301833	5 μl/10^6 cells
CD56	PE	HCD56	Biolegend	Mouse IgG1,κ	318305	5-20 μl/10^6 cells
CD45	PerCP	HI30	Biolegend	Mouse IgG1,κ	304025	5 μl/10^6 cells
CD16	BUV395	3G8	BD Bioscience	Mouse IgG$_1$,κ	563785	5 μl/10^6 cells
CD8ß	PE-Cy7	SIDI8BEE	Thermo Fisher	Mouse IgG1,κ	25-5273-41	5 μl/10^5 to 10^8 cells
CD19	BV785	SJ25C1	Biolegend	Mouse IgG1,κ	363027	5 μl/10^6 cells
CD4	FITC	SK3	Biolegend	Mouse IgG1,κ	344604	5-20 μl/10^6 cells
HLA-DR	AF700	L243	Biolegend	Mouse IgG2a,κ	307625	2 μg/10^6 cells
CD123	eF450	7G3	Thermo Fisher	Mouse IgG2a,κ	48-1238-42	5 μl/10^5 to 10^8 cells
CD11c	PE/Dazzle 594	3 9	Biolegend	Mouse IgG1,κ	301641	5 μl/10^6 cells
CD66b	AF647	G10F5	Biolegend	Mouse IgM,κ	305109	5 μl/10^6 cells
Fixable dye	Zombie NIR Fixable Viability		Biolegend		423105	
CD11b	BV650	ICRF44	Biolegend	Mouse IgG1,κ	301335	5 μl/10^6 cells

Supplementary table 1. Antibody details for flow cytometric analysis of joint aspirates.

Cell population	Parent population
B cells	non-granulocytes
$CD3^+$ cells	non-granulocytes
T_h cells	non-granulocytes
T_h cells	$CD3^+$ cells
T_h cells	T_h and T_k cells
T_k cells	non-granulocytes
T_k cells	$CD3^+$ cells
T_k cells	T_h and T_k cells
T_h and T_k cells	non-granulocytes
T_h and T_k cells	$CD3^+$ cells
NKT cells	non-granulocytes
NKT cells	$CD3^+$ cells

Cell population	Parent population
NK cells	non-granulocytes
Monocytes	non-granulocytes
mMDSC	non-granulocytes
all DCs	non-granulocytes
mDC	non-granulocytes
mDC	all DCs
pDCs	non-granulocytes
pDCs	all DCs
Neutrophils	all granulocytes
Eosinophils	all granulocytes
Basophils	all granulocytes

Supplementary table 2. Flow cytometric parameters which were used for estimation of the mean misclassification error for different classification methods.

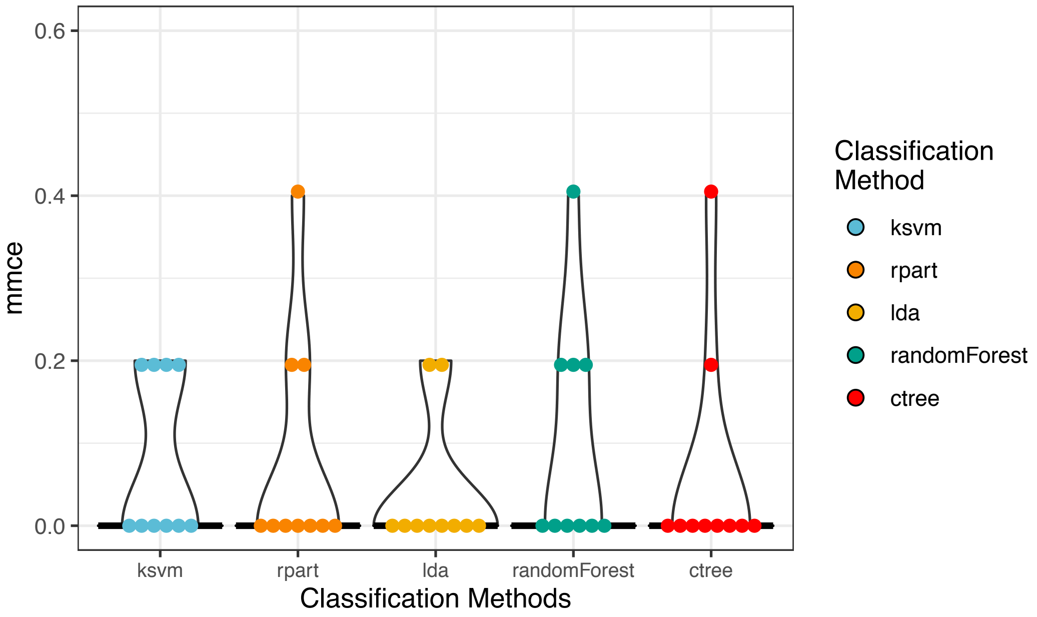

Supplementary figure 1. Estimation of the mean misclassification errors for 5 classification methods. The mean misclassification errors (mmce) for the five classification methods and 24 predictor variables (kvsm: Support Vector Machines, rpart: Decision Tree, lda: Linear Discriminant Analysis, randomForest: Random Forest, ctree: Conditional Inference Trees) are depicted as violin plots for all resampling iterations. Resampling strategy: 10-fold cross-validation. Each point represents an iteration.
